# Structures of *Helicobacter pylori* Shikimate Kinase Reveal a Selective Inhibitor-Induced-Fit Mechanism

**DOI:** 10.1371/journal.pone.0033481

**Published:** 2012-03-16

**Authors:** Wen-Chi Cheng, Yen-Fu Chen, Hung-Jung Wang, Kai-Cheng Hsu, Shuang-Chih Lin, Tzu-Jung Chen, Jinn-Moon Yang, Wen-Ching Wang

**Affiliations:** 1 Institute of Molecular and Cellular Biology and Department of Life Sciences, National Tsing Hua University, Hsinchu, Taiwan; 2 Biomedical Science and Engineering Center, National Tsing Hua University, Hsinchu, Taiwan; 3 Institute of Bioinformatics and Systems Biology, National Chiao Tung University, Hsinchu, Taiwan; 4 Department of Biological Science and Technology, National Chiao Tung University, Hsinchu, Taiwan; Institut Pasteur Paris, France

## Abstract

Shikimate kinase (SK), which catalyzes the specific phosphorylation of the 3-hydroxyl group of shikimic acid in the presence of ATP, is the enzyme in the fifth step of the shikimate pathway for biosynthesis of aromatic amino acids. This pathway is present in bacteria, fungi, and plants but absent in mammals and therefore represents an attractive target pathway for the development of new antimicrobial agents, herbicides, and antiparasitic agents. Here we investigated the detailed structure–activity relationship of SK from *Helicobacter pylori* (HpSK). Site-directed mutagenesis and isothermal titration calorimetry studies revealed critical conserved residues (D33, F48, R57, R116, and R132) that interact with shikimate and are therefore involved in catalysis. Crystal structures of HpSK·SO_4_, R57A, and HpSK•shikimate-3-phosphate•ADP show a characteristic three-layer architecture and a conformationally elastic region consisting of F48, R57, R116, and R132, occupied by shikimate. The structure of the inhibitor complex, E114A•162535, was also determined, which revealed a dramatic shift in the elastic LID region and resulted in conformational locking into a distinctive form. These results reveal considerable insight into the active-site chemistry of SKs and a selective inhibitor-induced-fit mechanism.

## Introduction

In recent years, major problematic bacterial infections have been described for methicillin-resistant *Staphylococcus aureus*, *Enterococcus faecium*, *Staphylococcus pneumonia*, *Klebsiella* species, *Acinetobacter baumannii*, *Pseudomonas aeruginosa*, *Mycobacterium tuberculosis*, and *Escherichia coli* [1]–[3]. The high prevalence of resistant bacteria and complex resistance, including multidrug-resistant pathogens, has been of particular concern. There are, however, limited antibacterial therapeutic options for the treatment of nosocomial infections for multidrug-resistant Gram-negative bacteria [4]. Health professionals are very aware of the urgent need for novel antibiotic agents against Gram-negative bacteria [5]. Despite a growing need for new and more effective antibiotics (or other means) to cure problematic bacterial infections, few new agents have been found in recent years owing to a substantial decline in research and development investment in the face of a challenging economic climate [6], [7].

The shikimate pathway is comprised of seven enzymatic components that convert erythrose 4-phosphate and phosphoenolpyruvate into chorismate, for subsequent synthesis of aromatic compounds [8]. This pathway is present in microbial cells, apicomplexan parasites, and plants but is absent in animals; this makes it an attractive target pathway for the development of new antimicrobial agents, herbicides, and antiparasitic agents [9]–[18]. Of note, 5-enolpyruvylshikimate 3-phosphate synthase (the sixth enzyme in the shikimate pathway) has been successfully targeted with glyphosate, one of the world's best-selling herbicides [19], [20]. Disruption in *M. tuberculosis* of *aroK*, the gene encoding shikimate kinase (SK, EC 2.7.1.71), the fifth enzyme of the shikimate pathway, further suggests that this pathway is essential for antimicrobial drug discovery [21].

SK catalyzes the specific phosphorylation of the 3-hydroxyl group of shikimic acid, using ATP as a co-substrate. Several SK structures are available (from *E. coli*, *Erwinia chrysanthemi*, *Campylobacter jejuni*, *Aquifex aeolicus* and *Arabidopsis thaliana* [22]–[27]), and also from two important pathogens, *M. tuberculosis* and *Helicobacter pylori* (MtSK and HpSK, respectively) [28]–[33]. SKs belong to a class of P-loop kinases that share a homologous α-β-α fold [23], [34]. These structures have an active site created by conserved residues and occupied by ATP and shikimate. The occupancy of this site by substrates/products is associated with inducing an open-to-closed conformational change by a flexible loop, and domain movement for SKs [32]. Such movement, as is the case for many other kinases, is essential for catalytic turnover [34]. Understanding the critical residues involved in ligand binding and conformational flexibility is therefore essential in aiding design of potential selective inhibitors [35], [36].

The likelihood of HpSK as a target enzyme for potential drug and herbicide discovery prompted us to investigate the detailed structure-activity relationship of the binding pocket. Here, we report the crystal structures of HpSK·SO_4_, R57A, and HpSK• shikimate-3-phosphate (S3P)•ADP, which reveal that three conserved Arg residues (R57, R116, R132), the side chain of D33, and the aromatic ring of F48 are involved in binding to shikimate. We also determined the X-ray structure of the E114A mutant SK-inhibitor complex using a selective inhibitor (NSC162535; IC_50_ = 4.9 µM) identified from virtual docking analysis. Site-directed mutagenesis and isothermal titration calorimetry (ITC) together revealed the key binding residues and a NSC162535/induced-fit mechanism.

## Results

### Site-directed mutagenesis of shikimate-binding residues

One strategy to derive a specific selective inhibitor toward a given P-loop kinase is to target the non-ATP-binding site, because P-loop kinases possess a relatively conserved ATP site that catalyzes the phosphotransfer reaction [34]. To this end, we evaluated the shikimate-binding (SB) residues of HpSK. Structural comparison of reported SKs show that the structures are mostly homologous and contain a binding pocket consisting of nucleotide and shikimate sites [22]–[27]. The most significant structural deviation between the different structures is found in the LID region, where an open/closed structural change occurs upon ligand binding (Fig. S1). Based on the HpSK·shikimate·PO_4_ structure (1ZUI) [33], shikimate binds to residues from three subsites: (i) C_X_, where a carboxyl moiety of shikimate makes contact with R57, R116, and R132; (ii) O_CORE_, where two hydroxyl groups of shikimate make contact with M10, D33, G79–G81, and E114; and (iii) O_LID_, where a *trans* hydroxyl group of shikimate interacts with V44, F48, E114, and R116. Of these residues, D33, R57, G79–G81, R116, and R132 are strictly conserved among all SKs, whereas the others (M10, V44, F48 and E114) are relatively conserved (Fig. S2). Superposition analysis showed that these residues essentially overlap, except for M10 and E114. We therefore chose the following residues for site-directed mutagenesis studies: strictly conserved residues (D33, R57, R116, and R132) and moderately conserved residues (M10, F48, and E114). Each of these sites was replaced with Ala or a more conservative amino acid, as indicated in Table 1, and the resulting mutant proteins were expressed in *E. coli*. After purification by affinity chromatography, all mutants were analyzed by SDS-PAGE; each migrated as a major band of apparent molecular mass of approximately18 kDa, as expected. Each of the purified proteins was subjected to size-exclusion chromatography. This gave a single and sharp peak, suggesting each mutant existed in a compact globular form.

**Table 1:** Comparison of the relative activities and *K_d_* values of wild-type and mutant HpSK.

Mutants	Relative activity (%)	*K_m_* (µM) [ATP/SKM]	*K_d_* (µM)	
			shikimate	NSC162535
WT	100	101/60	0.32±0.07	8.7±0.9
M10A	38	293/135	34±7	2.8±0.8
D33A	ND		ND	9±2
D33E	ND		ND	14±3
F48A	ND		ND	ND
F48Y	40	231/291	5.2±0.4	12±2
R57A	2		ND	ND
R57K	2		ND	48±31
E114A	82	143/39	0.33±0.03	11±4
R116A	ND		ND	9±2
R116K	ND		ND	4.8±0.6
R132A	5		ND	ND
R132K	ND		ND	ND

WT, wild type; ND, not detectable.

We also performed differential scanning calorimetry (DSC) experiments to evaluate the stability and unfolding of each of the generated HpSK mutants. All HpSKs revealed a single transition and fit well (Fig. S3). Wild-type HpSK showed a single transition peak upon thermal unfolding, revealing a Tm value of 47°C (Table S1). The other HpSK mutants also demonstrated a single peak and had comparable Tm values (between 44°C and 50°C), except for M10A (Tm = 55°C) and D33A (Tm = 41°C). It is noted that purified D33A formed some precipitate after 1–2 weeks, in line with a reduced thermal stability observed in the DSC results.

Enzymatic analysis of R57A, R116A and R132A revealed that these mutants completely lost enzyme activity (Table 1), suggesting that R57 and R132, from the C_X_ site, are important in catalysis. R116 is also noted to contribute to both the C_X_-site and O_LID_-site interactions. Importantly, it has been suggested that R117 of MtSK (corresponding to R116 of HpSK) may be the primary residue involved in stabilizing the pentavalent phosphorus transition-state intermediate [31]. Replacing Arg with Lys at these sites (R57K, R116K, and R132K) did not restore any detectable activity (Table 1). These results together suggest the importance of three Arg residues in catalysis. F48 is located in the O_LID_ subsite and the mutant F48A exhibited no detectable activity, although F48Y restored activity to 40%, indicating that the aromatic ring of F48 contributes to catalytic activity. In support of this view, we note that the aromatic ring of F48 makes contact with R57 and other nearby residues (V44, E53, F56, and P117) and forms stable interactions, thus ensuring R57 is in an appropriate position to interact with shikimate. The side chain of the other O_LID_ residue, E114, faces the solvent, and E114A retained 82% relative activity. For O_CORE_ residues, D33A and D33E showed loss of enzymatic activity, whereas M10A retained 38% relative activity.

We also investigated the kinetics of three mutants (M10A, F48Y and E114A) that retained enzymatic activity. The apparent values for Michaelis-Menten parameters were determined as follows: M10A, *K_m_*_ (MgATP)_ = 293±42 µM, *K_m_*_ (shikimate)_ = 135±26 µM, *V*_max(MgATP)_ = 26±1 µmol/min/mg, *V*_max(shikimate)_ = 16±1 µmol/min/mg; F48Y, *K_m_*_(MgATP)_ = 231±35 µM, *K_m_*_(shikimate)_ = 291±99 µM, *V*_max(MgATP)_ = 17±1 µmol/min/mg, *V*_max(shikimate)_ = 13±2 µmol/min/mg; E114A, *K_m_*_(MgATP)_ = 143±18 µM, *K_m_*_(shikimate)_ = 39±7 µM, *V*_max(MgATP)_ = 26±2 µmol/min/mg, *V*_max(shikimate)_ = 23±1 µmol/min/mg. As compared with the wild-type enzyme (*K_m_*_(MgATP)_ = 101±17 µM, *K_m_*_(shikimate)_ = 60±8 µM, *V*_max(MgATP)_ = 26±1 µmol/min/mg, *V*_max(shikimate)_ = 22±1 µmol/min/mg) [33], M10A and F48Y exhibited lower affinity toward MgATP and shikimate, respectively, in line with their lower relative activities (Table 1). For the LID mutant E114A, it displayed very similar kinetics as that of wildtype enzyme; there was only a slightly lower affinity toward MgATP.

The crystal structure shows that D33 forms a hydrogen bond with the 3-OH group of shikimate, which may increase the nucleophilicity of the O atom or accept the proton from the 3-OH group of shikimate. M10, on the other hand, shows limited contacts with shikimate; thus, replacement with Ala at this site did not completely eliminate the enzymatic activity. Together, our results suggest that D33, F48, R57, R116, and R132 are critical for enzymatic catalysis.

### Comparison by ITC of the association between wild-type or mutant HpSKs with shikimate or the inhibitor, NSC162535

**Figure 1:**
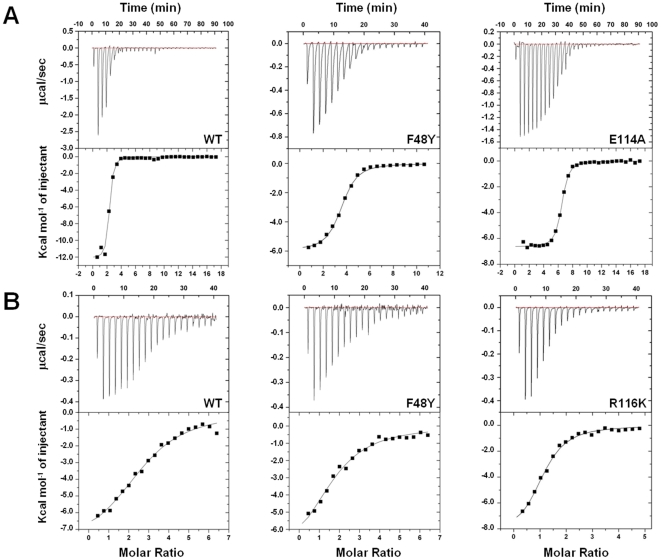
Binding properties of HpSK mutants. Isothermal titration calorimetry data showing, (A) the titration of shikimate into wild-type, F48Y, and E114A, and (B) the titration of NSC162535 into wild-type, F48Y, and R116K. In each panel, the upper portion shows raw data for the titration, and the lower portion shows the normalized, integrated binding isotherm together with the fitted binding curve.

Using GEMDOCK docking algorithms, we modeled a pocket that consists of the crucial SB residues (D33, F48, R57, R116, R132) involved in catalysis, based on the HpSK·shikimate·PO_4_ structure (1ZUI) [33] to search for putative inhibitors [37], [38] in the Maybridge and NCI databases. We were thus able to identify a potent competitive inhibitor, NSC162535 (IC_50_ = 4.9 µM; *K_i_* (shikimate) = 1.8 µM; *K_i_* (ATP) = 1.9 µM; Fig. S5). We next characterized the properties of those crucial residues for binding to shikimate, using the ITC experiments. For the wild-type HpSK (15 µM HpSK, 0.1 mM ADP, 0.5 mM Mg^2+^), a clear shikimate ITC pattern was observed, showing a high binding affinity to shikimate (*K_d_* = 0.32 µM; Fig. 1 and Table 1). By contrast, D33A, D33E, R57A, R57K, R116A, R116K, R132A and R132K displayed no heat release. For F48, F48A lost binding activity, whereas F48Y partially restored the shikimate binding activity relative to F48A (*K_d_* = 5.2 µM), revealing that the aromatic ring contributes towards binding to shikimate. Thus, the side chains of D33 and three Arg residues (R57, R116, R132) as well as the aromatic ring of F48 in HpSK are critically involved in shikimate binding (Fig. 1A). We also evaluated the binding properties of the two mutants, M10A and E114A, which exhibited a good portion of enzymatic activity. M10A had a relatively low affinity for shikimate (*K_d_* = 34 µM; Fig. S4A), whereas E114A had a shikimate binding affinity comparable to that of wild-type (*K_d_* = 0.33 µM; Fig. 1A).

We further utilized ITC to measure the binding of NSC162535 to each of the HpSK mutants (Fig. 1B, Fig. S4B, and Table 1). The wild-type enzyme had detectable binding affinity for NSC162535 (*K_d_* = 5.2 µM). With respect to the critical residues (D33, F48, R57, R116, R132), the mutants F48A, R57A, R132A, and R132K lacked affinity for NSC162535. F48Y, however, retained binding affinity (*K_d_* = 12 µM; Fig. 1B), whereas R57K had reduced affinity (*K_d_* = 48 µM), indicating that replacement with a more conservative side chain at F48 and R57 partially rescued the binding affinity. In contrast, the mutants D33A and D33E, and R116A and R116K had measurable ITC binding profiles with comparable *K_d_* values (Table 1 and Fig. S4B), suggesting that the D33 carboxyl moiety and the R116 guanidino group make a lesser contribution to binding of NSC162535. M10A and E114A also had measurable affinity (Table 1 and Fig. S4B). These results together suggest that side chains from R57 and R132, as well as the aromatic ring from F48, are most crucial in interacting with NSC162535, and that D33 and R116, which are important for binding to shikimate, contribute less to the interactions with NSC162535.

### Crystal structures of HpSK·SO_4_, HpSK• S3P•ADP and R57A

**Figure 2:**
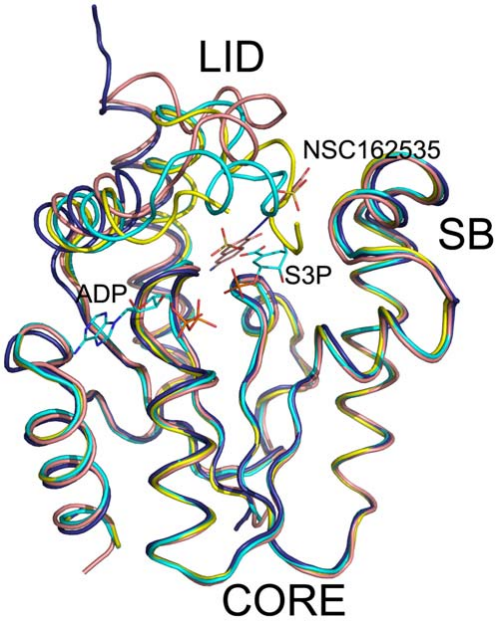
Superposition of four HpSK structures. The following structures are shown: HpSK·dimer·SO_4_ (blue), HpSK·shikimate·PO_4_ (1ZUI) (yellow), HpSK·S3P·ADP (cyan), and E114A·162535 (pink). These structures show homologous folds but a flexible LID segment.

**Figure 3:**
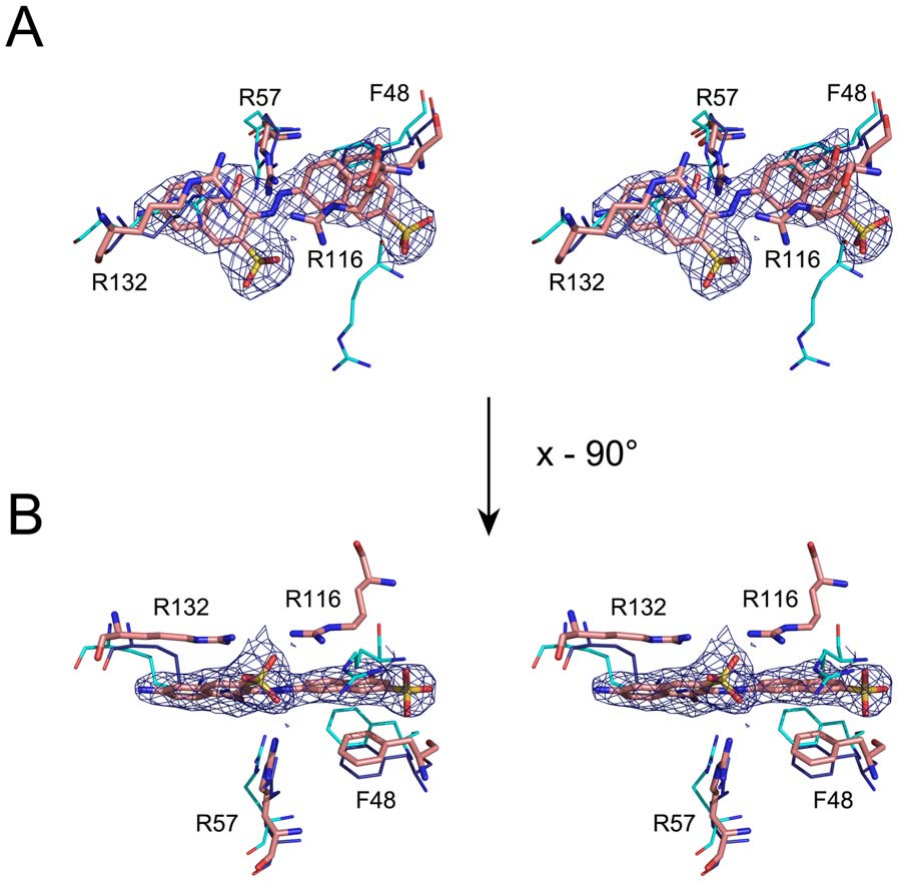
Stereoview of the electron density map of NSC162535. (B) is 90 degree rotation clockwise of (A). F48, R57, R116, and R132 are shown onto superimposed structures [E114A·162535 (pink), HpSK·dimer·SO_4_ (blue), and HpSK·S3P·ADP (cyan)]. The 2*Fo*−*Fc* electron density map shows the bound NSC162535, contoured at the 1.0-σ level.

Crystal structures of HpSK and MtSK have been reported, alone and in complex with either one or two substrates/products [28]–[33]. Based on several MtSK crystal structures, Hartmann *et al.* proposed a model for the random sequential binding of substrates (ATP and shikimate) associated with domain movements [32]. Here, we additionally determined structures of a dimeric HpSK·SO_4_ (R = 22.7%, R_free_ = 26.0%), HpSK• S3P•ADP (R = 23.1%, R_free_ = 27.7%), and R57A (R = 24.6%, R_free_ = 27.3%) (Fig. 2, 3, Fig. S6, and Table 2). Overall, these structures have a characteristic α-β-α fold that consists of the CORE domain (residues 1–31, 61–108, and 124–162), the SB domain (residues 32–60), and the LID region (residues 109–123) in the monomer [33] (Fig. 2). The LID region is most flexible and flips over the binding pocket, in an open or closed state [33]. For the dimeric HpSK·SO_4_ structure, the LID region points upward, and a number of residues cannot be built into this region (residues 108–118 in subunit A, and 111–117 in subunit B), implying a disordered region.

**Table 2:** X-ray data collection and refinement statistics.

	HpSK·dimer·SO_4_	HpSK·S3P·ADP	HpSK·R57A	E114A·162535
**PDB code**	3HR7	3MUF	3MRS	3N2E
**Data collection**				
Source	NSRRC BL-13B1^a^	NSRRC BL-13C1^a^	SPring-8 BL-12B2^a^	SPring-8 BL-12B2
Wavelength (Å)	1.000	0.9762	1.000	1.000
Temperature	−165°C	−165°C	−165°C	−165°C
Space group	C2	P61	P43212	C2
Cell dimensions				
*a*, *b*, *c* (Å)	122.48, 59.61, 80.76	98.82, 98.82, 42.13	88.90, 88.90, 39.87	193.91, 71.79, 47.63
α, β, γ (°)	90, 113.08, 90	90, 90, 120	90, 90, 90	90, 91.96, 90
Resolution range (Å)	30-1.80 (1.86-1.80)^b^	30-2.30 (2.38-2.30)	30-2.40 (2.49-2.40)	30-2.53 (2.62-2.53)
*R*_merge_ (%)^c^	6.8 (41.8)	6.8 (48.8)	3.8 (13.1)	5.3 (30.5)
*I*/σ*I*	18.0 (2.6)	29.3 (2.5)	56.0 (19.0)	26.8 (4.3)
Completeness (%)	98.2 (94.1)	99.9 (99.5)	99.8 (100.0)	99.7 (98.3)
Redundancy	3.8 (3.5)	11.7 (10.7)	10.4 (10.6)	5.1 (4.7)
**Refinement**				
Resolution (Å)	30.0–1.80	30.0–2.30	30.0–2.40	30.0–2.53
No. reflections	46,105	10,003	6,287	20,807
*R*_work_ d/*R*_free_ e	0.227/0.260	0.231/0.277	0.246/0.273	0.218/0.262
No. atoms				
Protein	2422	1279	1172	3661
Ligand/ion	10	43	0	116
Water	316	75	25	83
r.m.s. deviation^f^				
Bond length (Å)	0.017	0.018	0.016	0.022
Bond angle (°)	1.546	1.780	1.617	2.018
Overall B factor (Å^2^)				
from Wilson plot	27.50	36.00	44.20	40.00
from protein model	29.45	56.19	28.24	44.30
Ramachandran analysis (%)				
Favored	99.7	96.8	96.6	97.6
Allowed	0.3	3.2	3.4	2.4
Generous	0.0	0.0	0.0	0.0
Disallowed	0.0	0.0	0.0	0.0
Estimated coordinate error (Å)	0.117	0.242	0.537	0.292

All data sets were collected from a single crystal.

a BL-13B1/13C1 National Synchrotron Radiation Research Center (NSRRC), HsinChu, Taiwan; Taiwan BL-12B2 beamline at SPring-8, Hyogo, Japan.

b Values in parentheses refer to statistics in the highest-resolution shell.

c *R*_merge_ = ∑|*I*_obs_−<*I*>|∑*I*_obs_.

d *R* = ∑|*F*_obs_−*F*_calc_|/∑*F*_obs_, where *F*_obs_ and *F*_calc_ are the observed and calculated structure factor amplitudes, respectively.

e *R*_free_ was computed using 5% of the data assigned randomly.

f r.m.s., root mean square.

The HpSK•S3P•ADP structure shows clear electron density for all residues. Within the binding pocket, there is a large piece of non-peptide density that can easily be modeled as the product S3P, and nearby density can be built as ADP (Fig. 2). The final structure includes an ordered and complete LID segment that closes over the binding pocket, in accordance with the closed-form MtSK•S3P•ADP structure (2IYZ; root mean square deviation of Cα atoms = 1.26 Å) [32]. The LID region covers the binding pocket in which the guanidino group of R116 has direct contacts with S3P and the β-phosphate of ADP, forming strong hydrogen bonds. In the HpSK•shikimate•PO_4_ structure, R116 also makes a hydrogen bond with the carboxyl moiety of shikimate. Based on these structures, it is likely that S3P is chelated by R57 and R132 via a hydrogen-bonding network in HpSK•shikimate•PO_4_ and HpSK•S3P•ADP. This would lead to a small movement in the peptide backbone, propagated through the α6 helix into the adjacent LID loop. A subsequent large conformational rearrangement of this loop would allow the side chain of R116 to bind to the phosphate group of S3P, as seen in HpSK•S3P•ADP, or possibly the γ-phosphate moiety of ATP [32].

We also determined the structure of mutant R57A (Fig. S6 and Table 2) that lacked enzyme activity. In the wild-type HpSK, the guanidino group of R57 forms hydrogen bonds with the two carboxyl groups from E53 and E60. Replacement of the guanidino side chain with a methyl group at this pocket eliminates these interactions in R57A. Instead, E53 from the SB domain forms hydrogen bonds to the guanidino group from R132 (E53 [Oε1]-R132 [Nη1]: 2.21 Å; E53 [Oε2]-R132 [Nη2]: 3.58 Å) located at α6 (CORE). The E53–R132 proximity in R57A appears to induce a conformational move for the SB domain (residues 43–63; α3, the α3–α4 loop, and α4) and slightly influences the CORE domain.

### Crystal structure of E114A complexed to NSC162535 reveals an inactivation mechanism

In an effort to understand the detailed structure-activity relationship of this inhibitor at atomic resolution, we attempted crystallization trials using either wild-type HpSK or a mutant prepared in this investigation. After extensive trials, only the map of the E114A·162535 crystal showed a large piece of residual density in the binding pocket, which could be modeled as NSC162535 (Fig. 3 and Fig. S5). The final E114A·162535 crystal structure shows a trimeric assembly (R = 21.8%, R_free_ = 26.2%; Table 2).

**Figure 4:**
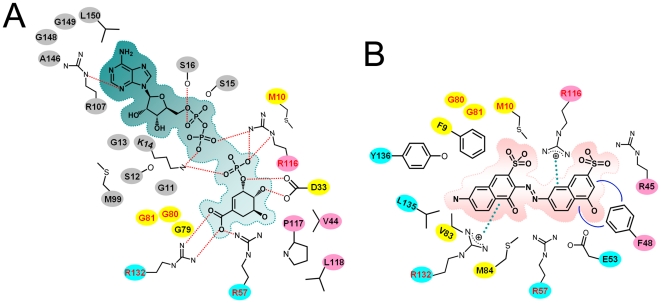
Schematic illustration of HpSK·S3P·ADP and E114A·162535 binding pockets. (A) Residues that contact S3P and ADP in HpSK·S3P·ADP. Red dashed lines denote hydrogen-bonding interactions. (B) Residues that contact NSC162535 in E114A·162535. Dotted green lines and blue curves are cation-π and π-π interactions, respectively. Residues from three HpSK·shikimate·PO_4_ subsites, C_X_, O_CORE_, and O_LID_ [33], are colored by cyan, yellow, and pink, respectively. Residues from the nucleotide site are colored by grey.

NSC162535, which is clearly observed in two of the three subunits, exhibits an extended conformation between LID and SB. Interestingly, it extends out to the entrance of the binding pocket (Fig. 2). The LID loop of HpSK accommodates the inhibitor by undergoing a large conformational switch, distinct from that of HpSK·SO_4_ and HpSK• S3P•ADP. Notably, the side chain of R116 at LID forms hydrogen bonds with an SO_4_ group and packs against one of the naphthalene moieties with an electron-rich π system (Table 3). This naphthalene group also packs against the aromatic ring of F48 from the SB domain on the other side, establishing strong cation-π and π-π interactions. The other naphthalene group interacts with the side chain of R132, yielding a cation-π interaction. Additionally, the guanidino groups of R116 and R57 are located near the diazo moiety of NSC162535, making cation-π interaction and a kind of polar interaction, respectively (Fig. 4B). This inhibitor also makes contact with residues from, or near, the Walker A (F9 and M10) and Walker B (G80, G81, V83, and M84) motifs, as well as with L135 and Y136 (Fig. 4B). Such strong cation-π, π-π, hydrogen-bonding interactions, and van der Waal contacts between NSC162535 and the surrounding residues induces a distinctive induced-fit conformation, as opposed to that seen in the binding pocket of HpSK• S3P•ADP (Fig. 4A). Superposition of HpSK• S3P•ADP and E114•162535 shows that only R57 and R132 stay at approximately the same position; M10 and R116 are situated at rather distinct positions to interact with S3P and NSC162535, respectively (Fig. 4 and Table 3). Additionally, HpSK•S3P•ADP and E114•162535 have different contacting residues, (D33, V44, G79, P117, L118, and F9, R45, F48, V83, M84, L135, Y136, respectively). Taken together, these data suggest a unique environment at this position, which explains its inhibitor selectivity (Fig. 4 and Table 3).

**Table 3:** Binding of shikimate-3-phosphate and NSC162535 to HpSK·S3P·ADP and E114A·162535.

Atom	S3P	Distance (Å)	Atom	NSC162535	Distance (Å)
HpSK·S3P·ADP			E114A·162535*		
C_X_					
			Glu53 Oε1	CAP	3.52
Arg57 Nη1	O4	3.29			
Arg57 Nη2	O4	3.37	Arg57 Nη2	OAL	3.16
				NAM	3.58
				NAN	3.28
			Arg132 Cβ	NAG	3.48
			Arg132 Cγ	CAE	3.3
				CAF	3.2
				NAG	3.33
			Arg132 Cδ	CAD	3.27
				CAE	3.15
				CAF	3.52
			Arg132 Nε	CAB	3.54
				CAC	3.4
				CAD	3.49
Arg132 Cζ	O4	3.55	Arg132 Cζ	CAC	3.47
	O5	3.36		CAK	3.53
Arg132 Nη1	C7	3.47	Arg132 Nη1	CAI	3.51
	O4	3.6		CAJ	3.5
	O5	2.61			
Arg132 Nη2	C7	3.4			
	O4	2.71			
	O5	3.36			
			Leu135 Cγ	NAG	3.46
			Leu135 Cδ2	NAG	2.9
			Tyr136 Oη	CAE	3.58
				CAH	3.54
O_LID_					
			Arg45 Cγ	OBA	3.17
				OBC	3.13
			Phe48 Cε1	CAQ	3.56
Arg116 Cγ	O7	3.48			
Arg116 Cδ	O7	3.53			
Arg116 Nε	O7	2.53			
Arg116 Cζ	O7	3.13	Arg116 Cζ	CAT	3.58
			Arg116 Nη1	OBG	3.26
Arg116 Nη2	O7	3.02	Arg116 Nη2	OBG	3.15
Pro117 Cδ	O3	3.54			
Leu118 Cδ1	O4	3.31			
O_CORE_					
			Phe9 Cδ1	OBE	3.55
			Met10 N	OBE	3.46
Met10 Cγ	O7	3.4			
Lys14 Nζ	O8	2.74			
Asp33 Cγ	O2	3.59			
Asp33 Oδ1	O2	3.04			
Asp33 Oδ2	C4	3.36			
	O1	3.17			
	O2	3.35			
	O6	3.45			
Gly79 Cα	O1	3.47			
	O2	3.5			
Gly80 N	C2	3.17			
	O1	3.09			
	O8	2.99			
Gly80 Cα	C2	3.45	Gly80 Cα	OBE	3.32
	O5	3.1		OBF	3.46
	O8	3.52			
Gly80 C	O5	3.13	Gly80 C	CAH	3.46
			Gly80 O	CAH	3.48
Gly81 N	C7	3.35			
	O5	3.19			
Gly81 Cα	O4	3.56	Gly81 Cα	CAC	3.59
	O5	3.59			
			Val83 Cγ1	NAG	3.42
			Met84 Cε	CAA	3.53
				CAB	2.93
				OAL	3.54

* Atom notation is given in Figure S5.

### A distinctive induced-fit conformational change of the inhibitor complex

Superposition of various structures (HpSK•SO_4_ open form; HpSK•shikimate•PO_4_, PDB code 1ZUI [33]; HpSK•S3P•ADP and E114A•162535) reveals a significant conformational change in the LID-containing segment after the β4 region of the CORE domain (residues 101–138; α5, LID and α6 regions; Fig. 2). Furthermore, the SB region (residues 32–60) shows a small rotation in the different liganded/non-liganded states, in agreement with the MtSK structure [32].

**Figure 5:**
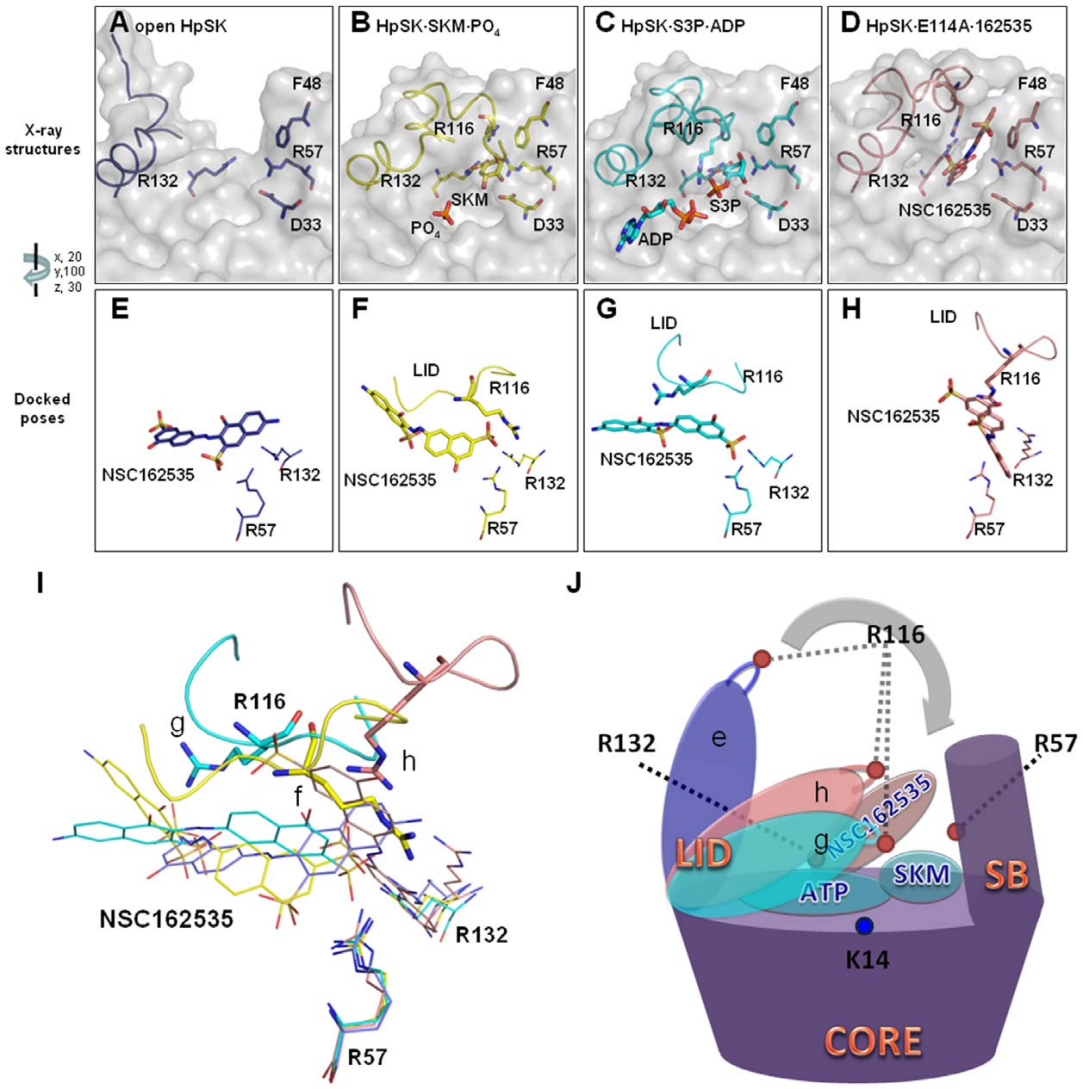
Probing the binding pockets in HpSK. (A–D) The binding pockets of HpSK: (A) open HpSK (3HR7), (B) HpSK·shikimate·PO_4_ (1ZUI), (C) HpSK·S3P·ADP (3MUF), and (D) E114A·162535 (3N2E). The bound ligands, D33, F48, R57, R116, and R132 are drawn as sticks. The LID segments (residues 109–123) are drawn as ribbon structures. (E–H) The docked NSC162535 models in the binding pockets of HpSK: (E) open HpSK, (F) HpSK·shikimate·PO_4_, (G) HpSK·S3P·ADP, and (H) E114A·162535. Superposition of the three residues R57, R116, and R132, docked and bound to NSC162535 with open HpSK (blue), HpSK·shikimate·PO_4_ (yellow), HpSK·S3P·ADP (cyan), and E114A·162535 (pink). (I) Superimposed docked structures (E–H). The conformation of the LID segment (residues 113–119, shown as ribbon) and R116 (thick stick) demonstrates the greatest conformational changes induced by bound ligands. (J) Schematic diagram of induced-fit conformational changes upon ligand binding. The view of LID regions corresponding to open HpSK (e), HpSK·S3P·ADP (g), and E114A·162535 (h) are colored as blue, cyan, and pink, respectively.

Of the three conserved Arg residues (R57, R116, R132), the Cα atom of R57 superimposes relatively well, whereas that of R132 has a small shift in the various structures (Fig. 5A–5D). Notably, there is a significant shift for R116 owing to the distinct conformations of the LID loop (Fig. 5A–5D). Our results suggest that these Arg residues contribute to the movement of the LID region and the SB domain upon binding to shikimate. R116, when visible, makes a significant shift to contact various ligands in the binding pocket: (i) shikimate in HpSK•shikimate•PO_4_; (ii) β-phosphate of ADP in HpSK•S3P•ADP; and (iii) NSC162535 in E114A•162535. In the MtSK•shikimate•AMPPCP structure (PDB code: 1ZYU [31]), R117 (corresponding to R116 in HpSK) directly contacts the γ-phosphate group of AMPPCP, an ATP analog, which supports its catalytic role in the γ-phosphoryl transfer [31].

To evaluate whether NSC162535 would come in contact with R116 in various conformations, we docked NSC162535 into each of the HpSK binding pockets (Fig. 5E–5I). In the open form, HpSK (flexible LID) presents a wide-opening pocket, allowing entry of promising substrates (Fig. 5A, 5E, and 5J). No close contacts were found between R116 and the docked NSC162535 in the binding pockets of the open and HpSK•shikimate•PO_4_ forms (Fig. 5E and 5F). In the HpSK•S3P•ADP state, NSC162535 docked into a site where there were no direct contacts with the Nη1 and Nη2 atoms of R116. NSC162535, on the other hand, docked into a comparable site in the E114A•162535 form; it directly contacts the Nη1 and Nη2 atoms of R116, demonstrating a distinctive induced-fit conformation.

## Discussion

In this investigation, we compared the shikimate binding properties of wild-type and mutant HpSKs using ITC. We identified three conserved Arg residues (R57, R116, R132) critical to interactions with shikimate, and therefore catalytic activity. Additionally, side chains from D33 and F48 were found to play important roles in binding to shikimate. Based on virtual docking analysis, we were able to design a potent inhibitor, NSC162535, for this SB pocket, which included these residues. Furthermore, we solved four HpSK structures: HpSK•SO_4_, HpSK•S3P•ADP, R57A, and E114A•162535.

Analysis of these structures reveals that R57 is critical for stabilizing and maintaining the optimal environment, whereas R132 plays a critical role in chelating the ligand. The R57A mutant structure shows a notable shift of the SB domain owing to the interactions between E53 and R132, rather than those between E53 and R57 in the wild-type structure, revealing a crucial role for R57 and R132 in defining the best active-site geometry to accommodate promising substrates (Fig. 3). In support of this view, R57 stays at a relatively identical site, whereas R132 shifts slightly among superimposed structures (Fig. 3, 5I and 5J).

R116, on the other hand, is located in the flexible LID segment and shows a significant move among various forms. It is visible in the complex structures with a closed LID loop (HpSK·shikimate·PO_4_, HpSK·S3P·ADP, and E114A·162535), where R116 directly contacts shikimate/S3P/NSC162535. Interestingly, ITC measurements revealed that this catalytic residue contributes substantially to shikimate binding but not to that of NSC162535. The insignificant contribution of R116 to NSC162535 binding (Table 1) implicates the likelihood that the R116-NSC162535 contacts, seen in the E114·162535 structure, result from induced conformational positioning.

Based on these results, we hypothesize that R57, in conjunction with R132, form an environment, along with F48, to interact with shikimate, triggering a cycle of conformational change. R116 sits at the LID segment and then contacts shikimate during the course of a conformational swap cycle to initiate catalysis of the specific phosphorylation of the 3-hydroxyl group (Fig. 5). Such positioning is crucial to yield an optimal conformation for R116 to stabilize the pentavalent transition-state intermediate [31], which allows catalysis to take place.

In regards to NSC162535 binding, it is likely that when NSC162535 interacts with R57 and R132, it also prompts a cycle of conformational change. The stable R57–R132 framework thereby provides an interacting anchor for not only shikimate/S3P but also NSC162535, triggering a conformational swap cycle to initiate a potential enzymatic reaction. Notably, the positioning optimization to trap NSC162535 yields a remarkably distinctive conformation, as seen in E114A•162535. Because there were nearly identical ITC binding data (either shikimate or NSC162535) between the wild-type and E114A, it is conceivable that the wild-type enzyme has an analogous binding configuration with NSC162535. As a result, the unusually elastic LID segment fits this inhibitor; R116 subsequently comes into contact with the SO_4_ group, locking into a distinctive induced form as seen in the complex structure.

## Materials and Methods

### Preparation of mutant HpSKs

Site-directed mutagenesis was performed using the overlap extension PCR method [39] with the plasmid pQE30-HpSK as the template. All mutations were confirmed by sequencing of the whole ligated PCR fragment. Mutant proteins were expressed and purified by the same procedures as based on published methods for HpSK [33].

### Enzymatic activity of wild-type and mutant HpSKs

SK activity was determined by coupling the release of ADP from the SK-catalyzed reaction to the oxidation of NADH using pyruvate kinase (EC 2.7.1.40) and lactate dehydrogenase (EC 1.1.1.27) as coupling enzymes. Shikimate-dependent oxidation of NADH was monitored by a decrease in *A*340 (ε = 6,200 M^−1^ cm^−1^). The assay was carried out at 25°C in a mixture containing 100 mM Tris-HCl, pH 7.5, 50 mM KCl, 5 mM MgCl_2_, 1.6 mM shikimic acid, 2.5 mM ATP, 1 mM phosphoenolpyruvate, 0.1 mM NADH, 2.5 U ml^−1^ pyruvate kinase, and 2.7 U ml^−1^ lactate dehydrogenase. All assays were conducted in a 96-well microplate and analyzed with a spectrophotometer (FLUOstar OPTIMA, BMG LABTECH). The relative activity of wild-type HpSK was set to 100%.

### Differential scanning calorimetry (DSC)

DSC measurements were carried out using a VP-DSC Microcalorimeter (Microcal, Northampton, MA). The HpSK proteins were in 40 mM Tris-HCl (pH 7.0) containing 100 mM NaCl. The DSC experiments were performed at a concentration of 0.1 mM HpSK protein. Prior to making measurements, a baseline was established by repeated scans of the sample cell containing only buffer solution. Scans were performed from low to high temperatures, at a rate of change of temperature of 1.0°C min^−1^. A buffer-buffer scan was subtracted from the buffer-sample scans, and linear-polynomial baselines were drawn for each scan. Baseline-corrected thermograms were then normalized to obtain the corresponding molar heat capacity curves. Midpoint temperature (Tm) values were estimated as the temperatures corresponding to the maximum of each thermogram peak. Analysis of DSC thermograms was implemented using Origin™ Software.

### Isothermal titration calorimetry (ITC)

Titration experiments were performed by ITC using an iTC200 or VP-ITC instrument (MicroCal, Piscataway, NJ, USA), and a 0.2-ml (iTC200) or 1.4-ml (VP-ITC) sample cell containing the macromolecule solution. All proteins were prepared in a buffer containing 20 mM potassium phosphate, pH 7.3. Prior to the experiment, samples were filtered and degassed under vacuum for 10 min in a Thermo Vac system (Microcal). The sample cell was filled with sample protein solution (15 µM) or the working buffer. Procedures of iTC200 titrations with a ligand (450 µM) were as follows: an initial 1-µl injection (not included in data analysis) followed by 19 injections of 2 µl each, with 2-min intervals between injections. Similar procedures for VP-ITC titrations were also performed: an initial 2-µl injection followed by 24 injections of 10 µl each, with 3-min intervals between injections. The experiments were performed with a constant stirrer speed of 1000 rpm (iTC200) or 290 rpm (VP-ITC) at 25°C. The binding isotherms were fitted to a one-site binding model to obtain the thermodynamic parameters with the initial point discarded. Data analysis was conducted using Origin 7 software.

### Crystallization and data collection

Crystallization was performed in 96-well microplates at 20°C using an Oryx8 robotic system (Douglas Instruments Ltd). The volume of HpSK protein solution (50 mg ml^−1^) in 40 mM Tris-HCl (pH 7.0) containing 100 mM NaCl was equal to the reservoir solution and equilibrated against 60 µl of reservoir solution. Initial crystallization conditions were screened using 672 different kit solutions (Hampton Research, Molecular Dimension and Jena Bioscience). We observed many different crystal forms in the drops.

The condition of HpSK·SO_4_ crystals was similar to that of apo HpSK crystals [33], which were obtained in buffer containing 0.2 M Li_2_SO_4_, 30% (w/v) polyethylene glycol (PEG) 8000, and 0.1 M sodium acetate buffer (pH 6.5). Crystals of HpSK·S3P·ADP, added to 5 mM shikimate and 5 mM MgATP, were obtained from 0.1 M HEPES sodium salt (pH 7.5), 0.1 M sodium acetate, 18% (w/v) PEG 8000 and 2% (w/v) 2-propanol. HpSK·R57A crystals were grown in a hanging-drop containing 0.1 M HEPES sodium salt (pH 8.0), 8% (w/v) 2-propanol and 18% (w/v) PEG4000. The best crystals of the E114A·162535 structure were obtained in a modified condition, containing 0.1 M HEPES sodium salt (pH 6.7) and 1.2 M potassium sodium tartrate tetrahydrate. Prior to data collection, crystals were dipped into Fomblin cryoprotectant oil for several seconds and then flash-frozen in a liquid nitrogen stream. The X-ray diffraction data were collected on NSRRC BL-13B1, BL-13C1, and SPring-8 BL-12B2 using an ADSC Quantum 4R CCD detector. All datasets were collected at −150°C and processed with the HKL/HKL2000 software suite [40]. Data collection statistics are shown in Table 2.

### Structure determination and refinement

The four structures were solved by molecular replacement with the program MOLREP [41] using the structure of the apo form of HpSK (PDB code, 1ZUH) as the search model. Further refinement was carried out using the maximum-likelihood target function embedded in the program REFMAC5 [42] and coupled to ARP/wARP [43]. Five percent of the reflections were randomly selected and used to compute a free R value (R_free_) for cross-validation of the model. 2*Fo−Fc* and *Fo−Fc* maps were produced and inspected after each cycle of refinement to revise the model manually on an interactive graphics computer with the program Coot [44]. The overall stereochemical quality of the final model was assessed with the program PROCHECK [45]. The atomic coordinates and structure factors were deposited in the RCSB Protein Data Bank with accession code 3HR7 (HpSK·SO_4_), 3MUF (HpSK·S3P·ADP), 3MRS (HpSK·R57A) and 3N2E (E114A·162535) (Table 2).

### Structural comparisons

Comparison with the six HpSK structures (PDB codes, 1ZUH [33], 1ZUI [33], 3HR7, 3MUF, 3MRS, 3N2E) and other SKs were carried out using the program LSQMAN in O [46] to superimpose Cα atoms, based on the optimized alignment. Structural figures were prepared with the program PyMOL.

### Molecular docking

The binding site for virtual docking screening of putative inhibitors was determined by considering the protein atoms located ≤10 Å from the SB site of MtSK (open-form structure; PDB code: 2IYT) and HpSK (open-form structure; PDB code: 1ZUH), respectively. We programmed GEMDOCK [38], [47]–[49] to screen Maybridge (65,947 compounds) and NCI (236,962 compounds) databases for both HpSK and MtSK. Top ranked compounds (n = 20) with the lowest energies were selected for testing in the enzyme inhibitory assay.

GEMDOCK was also used to dock NSC162535 to each of the four HpSK structures (HpSK·SO_4_, HpSK·shikimate·PO_4_, HpSK·S3P·ADP, and E114A·162535).

## Supporting Information

Figure S1 **Superposition of nine shikimate kinases.** The apo- and closed-HpSK (PDB codes: 1ZUH and 3MUF) are shown in blue and cyan, respectively. Apo form of MtSK (PDB code: 2IYT) is shown in orange, and closed-form (PDB code: 2IYQ) in yellow. The EcSK (PDB code: 1SHK) is red, and the EcSK complexed with ADP (PDB code: 2SHK) is pink. Green, brown and gray are indicated in CjSK (PDB code: 1VIA), EcoSK (PDB code: 1KAG) and AaSK (PDB code: 2PT5), respectively. (Hp: *Helicobacter pylori*; Mt: *Mycobacterium tuberculosis*; Ec: *Erwinia chrysanthemi*; Cj: *Campylobacter jejuni*; Eco: *Escherichia coli*; Aa: *Aquifex aeolicus*).
(TIF)

Figure S2 **Conserved residues of SKs and structure-based alignment of HpSK and MtSK.** Six shikimate kinases (*E. coli*, *E. chrysanthemi*, *C. jejuni*, *A. aeolicus*, *M. tuberculosis* and *H. pylori*) are aligned and shown with WebLogo program (http://weblogo.berkeley.edu/). HpSK and MtSK alignment are shown with ESPript program (http://espript.ibcp.fr/ESPript/ESPript/). The secondary structural elements are shown above the sequence. Mutants generated for the structure-activity analyses are indicated below the aligned sequence. Three arginines (R57, R116, and R132) belong to C_X_ shikimate-binding subsite. O_CORE_ consists of M10 and D33; and O_LID_ consists of F48, E114, and R116.
(TIF)

Figure S3 **DSC heat capacity curve of HpSK proteins.**
(TIF)

Figure S4 **Binding properties of HpSK mutants.** Isothermal titration calorimetry data showing (A) the titration of shikimate into M10A mutant; (B) the titration of NSC162535 into M10A, D33A, D33E, R57K, E114A and R116A.
(TIF)

Figure S5 **Chemical structure of NSC162535.**
(TIF)

Figure S6 **Conformational movement in the SB domain of HpSK·R57A structure.** A stereo view of the superimposed binding pocket between open HpSK (blue) and R57A (magenta) structures is shown. The oxygen, nitrogen and phosphorus atoms are colored red, blue and orange, respectively. The dashed line indicates hydrogen bonds.
(TIF)

Table S1 DSC thermodynamic parameters for the melting of HpSK wild-type and its mutants.
(DOC)
