# Supplementary figures and images for: Structures of Helicobacter pylori Shikimate Kinase Reveal a Selective Inhibitor-Induced-Fit Mechanism
Source: PLoS One. 2012 Mar 16;7(3):e33481. doi: 10.1371/journal.pone.0033481 (PMC3306394; doi:10.1371/journal.pone.0033481)

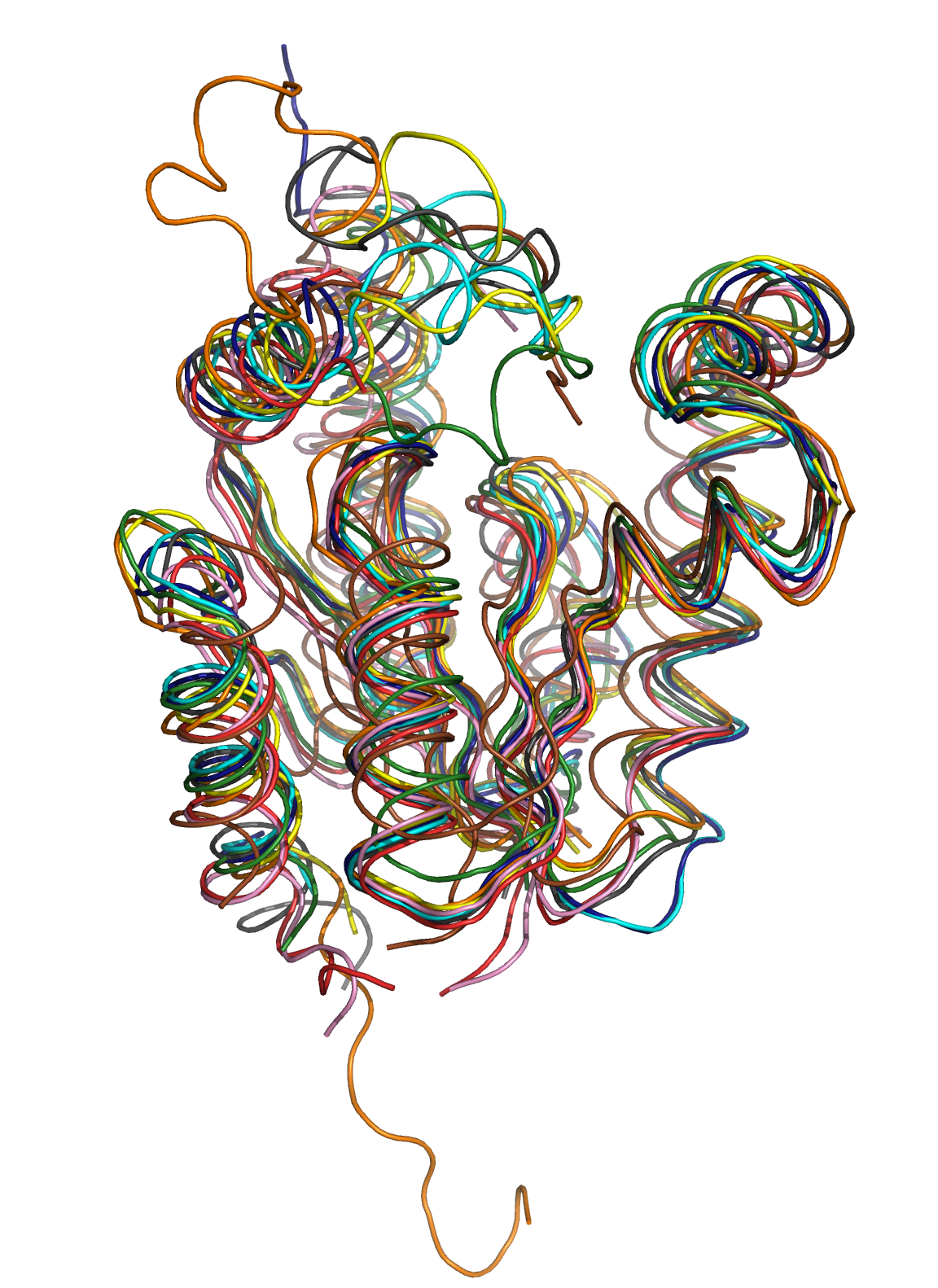

Supplement: Figure S1 — Superposition of nine shikimate kinases. The apo- and closed-HpSK (PDB codes: 1ZUH and 3MUF) are shown in blue and cyan, respectively. Apo form of MtSK (PDB code: 2IYT) is shown in orange, and closed-form (PDB code: 2IYQ) in yellow. The EcSK (PDB code: 1SHK) is red, and the EcSK complexed with ADP (PDB code: 2SHK) is pink. Green, brown and gray are indicated in CjSK (PDB code: 1VIA), EcoSK (PDB code: 1KAG) and AaSK (PDB code: 2PT5), respectively. (Hp: Helicobacter pylori; Mt: Mycobacterium tuberculosis; Ec: Erwinia chrysanthemi; Cj: Campylobacter jejuni; Eco: Escherichia coli; Aa: Aquifex aeolicus). (TIF) [file pone.0033481.s001.tif]

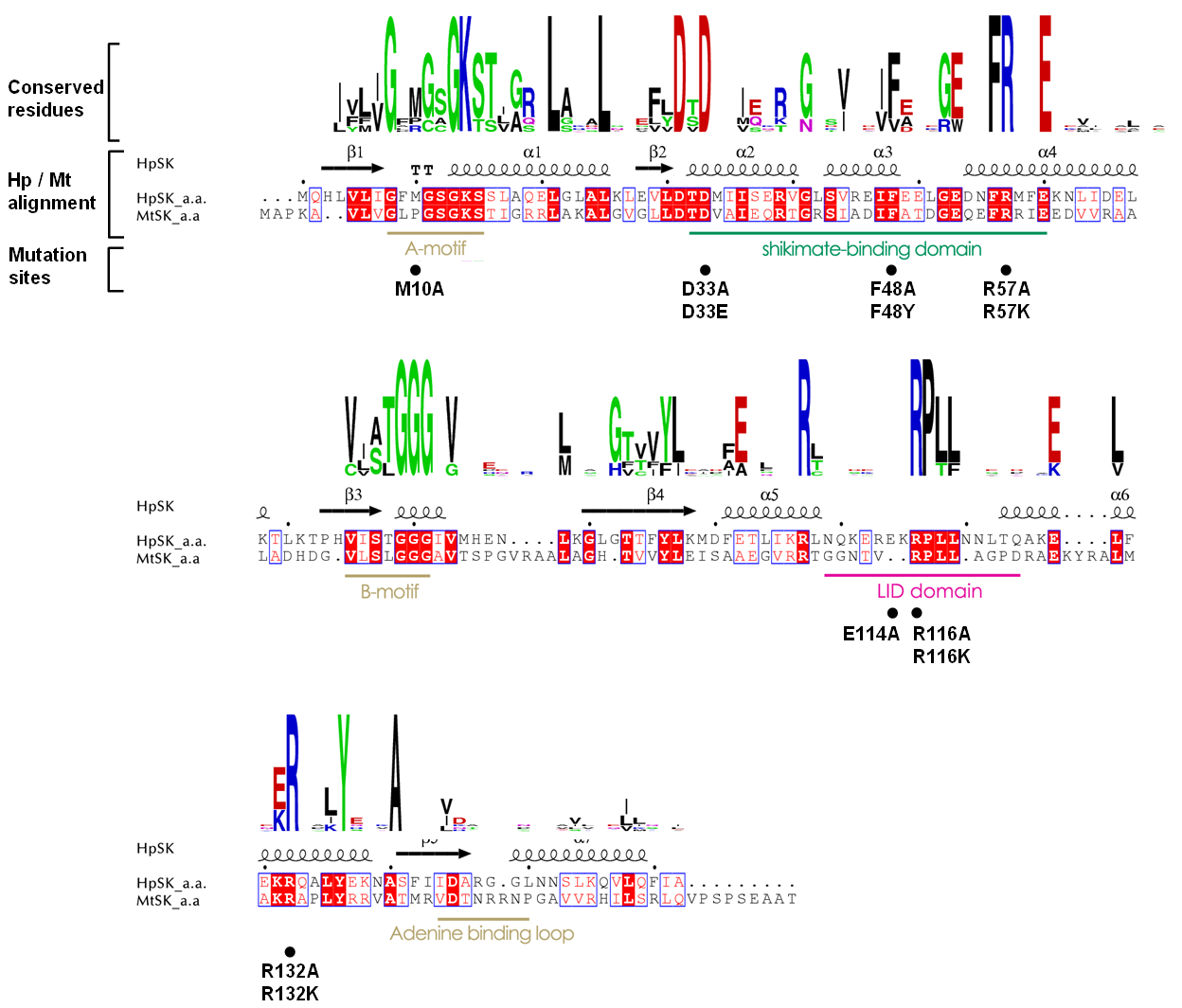

Supplement: Figure S2 — Conserved residues of SKs and structure-based alignment of HpSK and MtSK. Six shikimate kinases (E. coli, E. chrysanthemi, C. jejuni, A. aeolicus, M. tuberculosis and H. pylori) are aligned and shown with WebLogo program (http://weblogo.berkeley.edu/). HpSK and MtSK alignment are shown with ESPript program (http://espript.ibcp.fr/ESPript/ESPript/). The secondary structural elements are shown above the sequence. Mutants generated for the structure-activity analyses are indicated below the aligned sequence. Three arginines (R57, R116, and R132) belong to CX shikimate-binding subsite. OCORE consists of M10 and D33; and OLID consists of F48, E114, and R116. (TIF) [file pone.0033481.s002.tif]

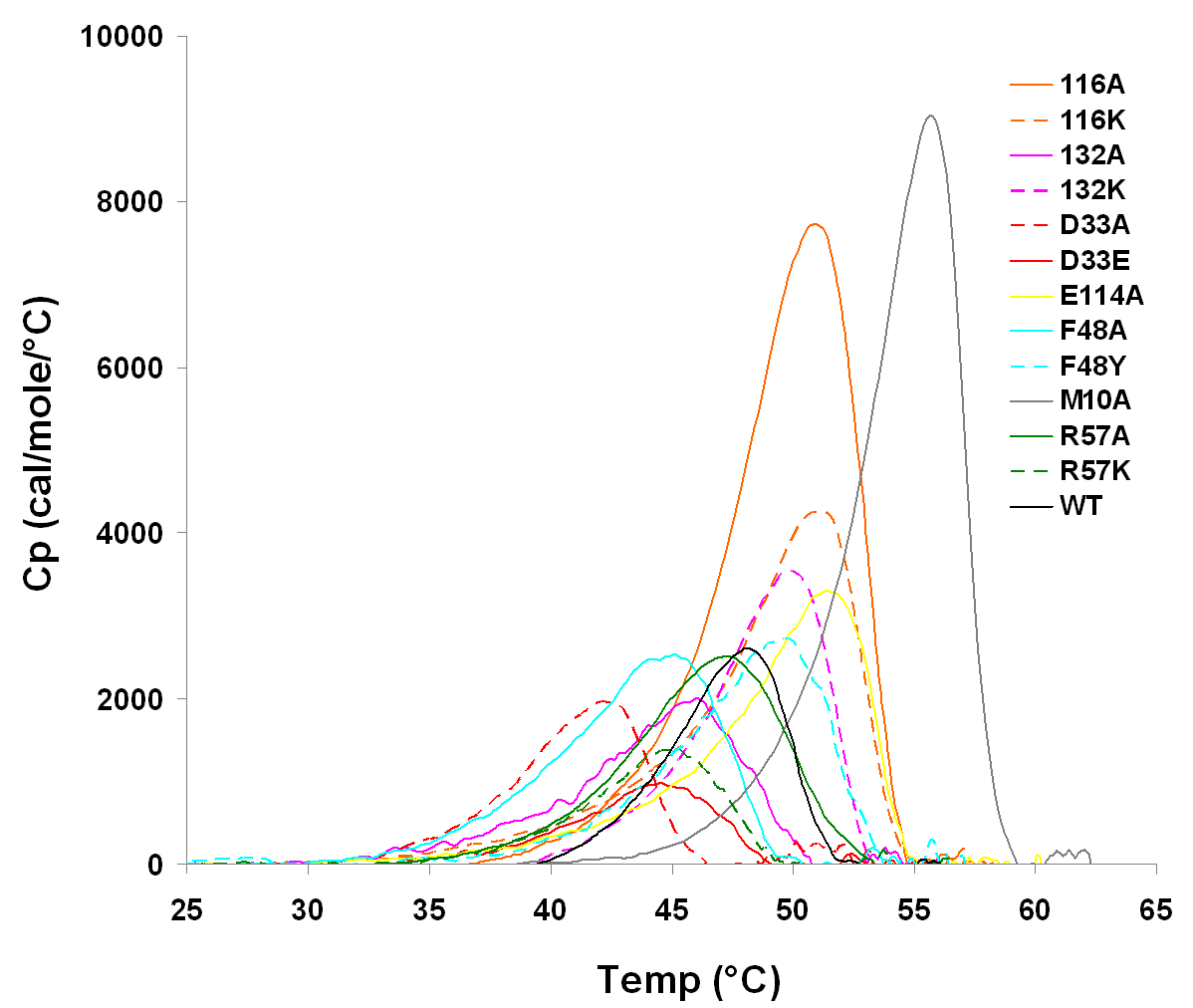

Supplement: Figure S3 — DSC heat capacity curve of HpSK proteins. (TIF) [file pone.0033481.s003.tif]

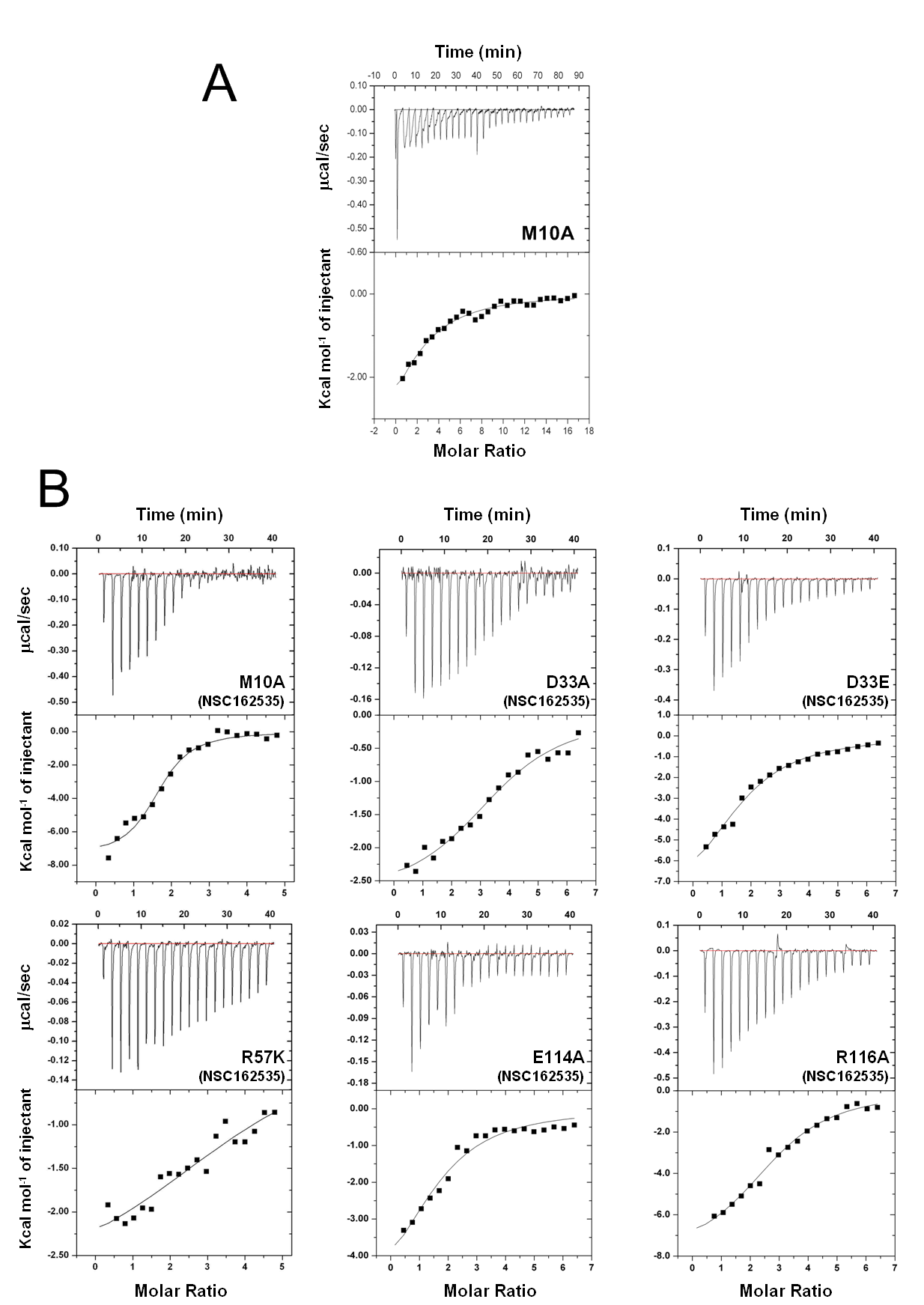

Supplement: Figure S4 — Binding properties of HpSK mutants. Isothermal titration calorimetry data showing (A) the titration of shikimate into M10A mutant; (B) the titration of NSC162535 into M10A, D33A, D33E, R57K, E114A and R116A. (TIF) [file pone.0033481.s004.tif]

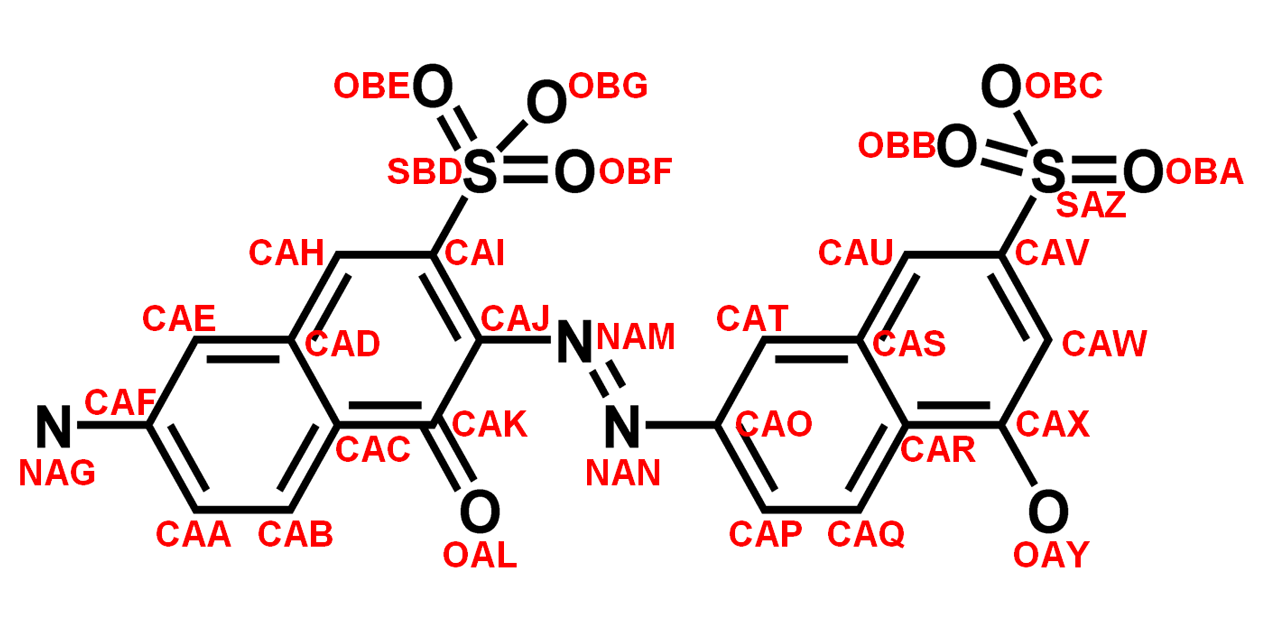

Supplement: Figure S5 — Chemical structure of NSC162535. (TIF) [file pone.0033481.s005.tif]

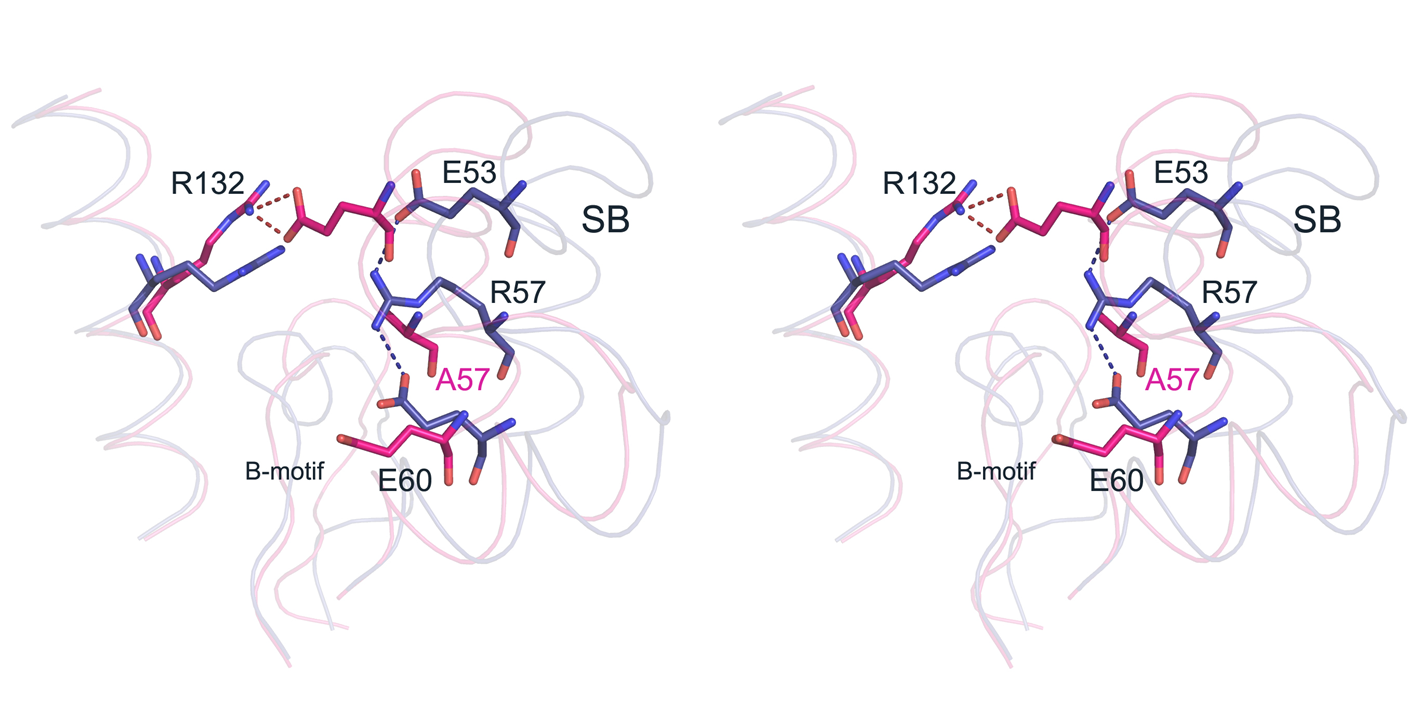

Supplement: Figure S6 — Conformational movement in the SB domain of HpSK·R57A structure. A stereo view of the superimposed binding pocket between open HpSK (blue) and R57A (magenta) structures is shown. The oxygen, nitrogen and phosphorus atoms are colored red, blue and orange, respectively. The dashed line indicates hydrogen bonds. (TIF) [file pone.0033481.s006.tif]
