# Supplementary material for: Structures of Helicobacter pylori Shikimate Kinase Reveal a Selective Inhibitor-Induced-Fit Mechanism
Source: PLoS One. 2012 Mar 16;7(3):e33481. doi: 10.1371/journal.pone.0033481 (PMC3306394; doi:10.1371/journal.pone.0033481)
Supplement: Table S1 — DSC thermodynamic parameters for the melting of HpSK wild-type and its mutants. (DOC) [file pone.0033481.s007.doc]

**Supplementary Table 1.** DSC thermodynamic parameters for the melting of HpSK wild-type and its mutants.

| Mutants | Tm (°C) | ∆H (cal/mol) | ∆H_V_ (cal/mol)^a^ |
| --- | --- | --- | --- |
| WT | 47 | 1.51E4 | 1.43E5 |
| M10A | 55 | 4.61E4 | 1.62E5 |
| D33A | 41 | 1.27E4 | 1.21E5 |
| D33E | 44 | 6.81E3 | 1.14E5 |
| F48A | 44 | 2.03E4 | 9.93E4 |
| F48Y | 49 | 1.98E4 | 1.10E5 |
| R57A | 47 | 1.98E4 | 1.04E5 |
| R57K | 46 | 9.25E3 | 1.21E5 |
| E114A | 50 | 2.22E4 | 1.19E5 |
| R116A | 50 | 4.89E4 | 1.31E5 |
| R116K | 50 | 2.87E4 | 1.19E5 |
| R132A | 45 | 1.52E4 | 1.00E5 |
| R132K | 49 | 2.10E4 | 1.38E5 |

^a^∆H_V_: Van’t Hoff enthalpy
